# Editorial: Understanding Crohn’s Disease: Immunity, Genes and Microbes

**DOI:** 10.3389/fimmu.2017.00357

**Published:** 2017-03-31

**Authors:** Amelia Sarmento

**Affiliations:** ^1^FP-ENAS/CEBIMED, Energy, Environment and Health Research Unit, Biomedical Research Centre, University Fernando Pessoa, Porto, Portugal; ^2^Instituto de Investigação e Inovação em Saúde, Universidade do Porto, Porto, Portugal; ^3^Instituto de Biologia Molecular e Celular (IBMC), Universidade do Porto, Porto, Portugal

**Keywords:** inflammatory bowel diseases, Crohn’s disease, genetic susceptibility, T cells, intestinal microbiota

**Editorial on the Research Topic**

**Understanding Crohn’s Disease: Immunity, Genes and Microbes**

Crohn’s disease (CD) is a chronic debilitating syndrome, associated with considerable morbidity and resulting in elevated public health costs every year. CD and ulcerative colitis (UC) are the two major forms of inflammatory bowel disease (IBD), which has long been characterized as an exacerbated inflammatory response to common antigenic stimuli in the gut due to immune dysregulation. Although both CD and UC share some common patterns, they are distinct diseases. In fact, while UC is characterized by a diffuse mucosal inflammation involving mainly the rectum and adjacent colonic tissue, CD is a transmural inflammatory disease that may involve any part of the gastrointestinal tract, from the mouth to the anus, although in most cases the terminal ileum is affected. Etiology of IBD, particularly CD, has been long debated and is likely to involve the contribution of multiple factors, making its study challenging. Among those contributing factors are genetic inheritance, epigenetic mechanisms, infection with particular pathobionts, and the gut microbiota in general. This topic aimed at bringing together contributions covering aspects related primarily to CD etiology and immunopathology, helping to drive forward a more comprehensive understanding of this challenging syndrome.

Genetic inheritance is an important predisposing factor for IBD. In a comprehensive review, Loddo and Romano point out the importance of genetic traits in susceptibility to IBD. The authors discuss the success of next-generation sequencing in the investigation of rare monogenic susceptibility variants, implicated in early-onset and very early-onset IBD. They also address the importance of epigenetic mechanisms, such as DNA methylation, in linking environmental stress and gene expression and discuss the use of microRNAs as biomarkers and therapeutical targets in IBD. The first genetic variant conferring susceptibility to ileal CD was located in the nucleotide oligomerization domain 2 (NOD2) gene (1), a gene implicated in recognition of bacterial muramyl dipeptide. Sidiq et al. highlight the role played by epithelial cell NOD2 expression in maintaining gut homeostasis and regulating ileal microbiota composition and address the consequences of a deficient NOD2 variant in gut dysregulation. As described in an original research article, Parkhouse and Monie tested whether loss-of-function NOD2 variants conferring susceptibility to CD (R702W, G908R, and L1007fsincC) exhibited deficient binding to receptor-interacting protein kinase 2, an adaptor protein implicated in NOD2 signaling. They found that impairment of NOD2 signaling shown by variants containing CD-susceptibility polymorphisms did not correlate with deficient RIP2 binding, concluding that the causes for impairment are multifactorial.

A set of articles included in this topic specifically relate to patterns of immune system function in CD. Di Giovangiulio et al. contributed with a comprehensive review on the neuromodulation of the gut immune system. Indeed, the cross talk between the nervous system and the gut immune system can shape the mucosal immune response to gut antigens and contribute to gut homeostasis. It is widely accepted that T cell function, particularly the interplay between regulatory and effector T cells, is of major importance in the maintenance of gut tolerance to intestinal antigens. Sarrabayrouse et al. discuss the important role played by a unique human subset of Treg cells, the IL-10-producers CD4CD8αα cells present in the intestinal mucosa. These cells are induced by clostridial bacteria to suppress T cell proliferation in a process independent on Foxp3 expression, since CD4CD8αα subset does not express this transcription factor. In a mini-review, Omenetti and Pizarro discuss the dynamic balance between intestinal Th17 and Treg cells, the plasticity allowing interdifferentiation and the participation of the gut microbiome in driving the differentiation toward each phenotype. Mucosal-associated invariant T cells (MAIT cells) are a non-conventional T cell subset possibly playing a role in CD (2). MAIT cells make up 10% of peripheral blood and intestinal lamina propria T cells and express Th17 cells markers. In an opinion article, Treiner elegantly discusses arguments in favor of the participation of MAIT cells in CD pathogenesis and presents two putative mechanisms by which activation of this T cell subset might occur in the gut.

In recent years, evidence is accumulating in favor of a central role of gut microbiota in CD pathogenesis. Three reviews included in this topic highlight the relevance of gut microbiota composition in health and disease. Haag and Siegmund discuss the interplay between intestinal microbiota and the innate immune system and the ways by which an altered microbiota may influence barrier integrity and activate innate immunity, leading to chronic inflammation. Buttó et al. also address current approaches available to investigate microbiota-associated mechanisms of disease, such as new animal models. Oberc and Coombes discuss the impact of antibiotic treatment and infectious gastroenteritis (two external risk factors for CD) on gut microbiota composition, leading to dysbiosis and consequently to disease onset. Sechi and Dow mini-review addresses the long debated participation of *Mycobacterium avium* subsp. *paratuberculosis* (MAP) in CD etiology, discussing MAP exposure, human genetic susceptibility to mycobacterial infection and MAP possible involvement in CD, and other human inflammatory diseases.

Finally, in line with increasing recognition of the role played by nutrition in disease prophylaxis and therapy, Ferguson discusses the beneficial effects of directed nutritional therapy for CD patients, according to the genetic background and presence of particular susceptibility polymorphisms. Although data on the benefits of particular nutrients already exist, systems biology approaches would allow to validate efficacy of particular diet constituents to CD patients. This would help to drive nutritional therapy into the primary therapy in CD, integrated with currently accepted approaches.

I hereby wish to manifest my appreciation to all the authors that participated in this research topic. Their articles significantly contributed to a more comprehensive view on CD immunopathology and associated factors, helping to improve current understanding of this debilitating disease.

## Author Contributions

The author confirms being the sole contributor of this work and approved it for publication.

## Conflict of Interest Statement

The author declares that the research was conducted in the absence of any commercial or financial relationships that could be construed as a potential conflict of interest.
